# The Effect of Adjunctive Systemic Antibiotics on the Outcomes of Non‐Surgical Periodontal Therapy: A Retrospective Cohort Study

**DOI:** 10.1111/jcpe.70117

**Published:** 2026-03-16

**Authors:** Georgios S. Chatzopoulos, Larry F. Wolff

**Affiliations:** ^1^ Department of Developmental and Surgical Sciences, Division of Periodontology, School of Dentistry University of Minnesota Minneapolis Minnesota USA; ^2^ Department of Periodontology, School of Dental Medicine Aristotle University of Thessaloniki Thessaloniki Greece

**Keywords:** amoxicillin–metronidazole, periodontitis, scaling and root planing, systemic antibiotics, treatment outcome

## Abstract

**Aim:**

To evaluate the real‐world comparative effectiveness of various adjunctive systemic antibiotic regimens in combination with scaling and root planing (SRP) using a large, multi‐centre retrospective cohort.

**Materials and Methods:**

Electronic health records were used retrospectively to categorise periodontitis patients into ‘SRP Only’ or ‘SRP + Antibiotic’ groups based on prescriptions within 7 days of therapy. Primary outcomes included changes in probing depth (PD), clinical attachment level (CAL), percentage of sites with PD ≥ 4 mm and bleeding on probing (BOP). Secondary outcomes assessed tooth‐level worsening in mobility or furcation status.

**Results:**

Among 3125 patients, the amoxicillin + metronidazole combination was found to be statistically superior to SRP alone for reducing PD, BOP and residual disease burden. It significantly reduced the odds of worsening tooth mobility by 63% and furcation involvement by 50%. While azithromycin and doxycycline improved CAL, the benefits of the amoxicillin + metronidazole combination were most pronounced in Stage III/IV (severe) periodontitis (−11.4% residual burden reduction, OR 4.55 for clinical success), compared to a modest effect in Stage I/II disease (−3.8%, *p* = 0.030).

**Conclusion:**

Adjunctive systemic antibiotics, particularly the amoxicillin + metronidazole combination, significantly enhance clinical outcomes in non‐surgical periodontitis treatment compared to SRP alone, with the greatest benefits seen in severe disease.

## 1 Introduction

Periodontitis is a chronic inflammatory disease initiated by a dysbiotic dental plaque biofilm, which leads to the progressive destruction of the tooth‐supporting tissues, including the periodontal ligament and alveolar bone (Sgolastra et al. 2012; Tonetti et al. 2018). The primary aetiology is bacterial, and therefore, the cornerstone of periodontal therapy is the mechanical disruption and removal of this biofilm through scaling and root planing (SRP) (Herrera et al. 2012). This procedure aims to eliminate plaque and calculus from the root surfaces, creating a biologically compatible environment for the periodontal tissues to heal. For many patients, SRP alone is effective in reducing inflammation, decreasing probing depth (PD) and achieving clinical attachment level (CAL) gain, thereby arresting the progression of the disease (Cobb 1996).

Despite the established efficacy of SRP, mechanical debridement has inherent limitations. Pathogenic bacteria can invade the adjacent soft tissues, reside in dentinal tubules or persist in anatomically complex areas like furcations and deep pockets, making them inaccessible to instrumentation alone (Socransky and Haffajee 1997; Zandbergen et al. 2013). Furthermore, pathogenic reservoirs on other oral surfaces, such as the tongue, tonsils and buccal mucosa, are not addressed by local mechanical therapy and can contribute to the recolonisation of treated periodontal pockets. The rationale for adjunctive systemic antibiotics is to target these residual, invasive and distant pathogens, thereby enhancing the clinical outcomes of mechanical therapy (Sgolastra et al. 2021; Haffajee et al. 2003).

The adjunctive use of systemic antibiotics, particularly the combination of amoxicillin and metronidazole, has been evaluated in numerous clinical trials and systematic reviews. Multiple meta‐analyses have consistently concluded that the use of these antibiotics in conjunction with SRP provides a statistically significant additional benefit in terms of PD reduction and CAL gain compared to SRP alone (Herrera et al. 2002; Sgolastra et al. 2012, 2021; Zandbergen et al. 2016). However, while the additional mean improvements—often in the range of 0.3–0.5 mm—may appear modest when averaged across entire patient populations, this focus on full‐mouth mean values can obscure more substantial benefits for specific patient subgroups. Furthermore, focusing solely on mean improvements is of limited clinical relevance, as the resolution of deep residual pockets is a primary goal of non‐surgical treatment and often dictates the need for further therapy (Herrera et al. 2012; Duarte et al. 2025). Concerns regarding potential adverse effects, the promotion of antibiotic resistance and outcome variability have led to a lack of consensus on their routine use (Preus et al. 2013).

The evidence for other systemic antibiotics, such as doxycycline or azithromycin, is less conclusive. One analysis found that systemic doxycycline did not have a significant impact on CAL, suggesting that the specific antimicrobial agent and its spectrum of activity are critical determinants of clinical success (Sun 2025). This underscores that a ‘one‐size‐fits‐all’ approach to systemic antibiotic use is inappropriate, and the choice of drug should be based on the strongest available evidence. This debate is further complicated by the considerable heterogeneity and methodological limitations within the existing literature from full‐mouth mean values across heterogeneous patient populations. This approach can mask the substantial benefits that may be confined to specific patient subgroups, such as those with more severe forms of periodontitis, thereby misrepresenting the potential value of targeted antibiotic therapy. The variability in patient populations (e.g., chronic vs. aggressive periodontitis), antibiotic protocols (dose and duration) and follow‐up periods makes it difficult to synthesise the data and draw definitive conclusions (Kondreddy et al. 2025).

This debate is complicated by the heterogeneity of existing literature, where variations in patient populations, antibiotic protocols and follow‐up periods make it difficult to draw definitive conclusions. Crucially, relying solely on full‐mouth mean values from randomised controlled trials (RCTs) can obscure substantial benefits confined to specific high‐risk subgroups, such as those with severe periodontitis. While RCTs have established efficacy in controlled settings, a significant gap remains regarding the effectiveness of these interventions in routine ‘real‐world’ clinical practice, where treatment is delivered by diverse providers to a heterogeneous patient population. Therefore, the aim of this study is to complement existing trial data by evaluating the comparative effectiveness of different antibiotic regimens—including head‐to‐head comparisons of monotherapies—in a large, retrospective cohort.

## 2 Materials and Methods

### 2.1 Study Design and Patient Cohort

**FIGURE 1:**
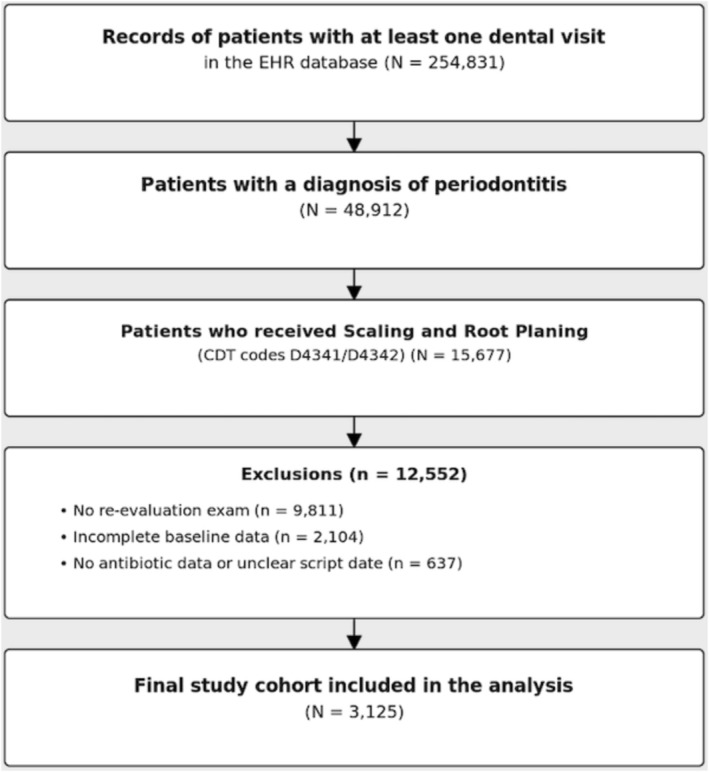
Flow diagram of cohort selection.

This retrospective cohort study utilised a large, de‐identified database of electronic health records (EHRs) from multiple university dental clinics within the BigMouth Consortium to analyse patients diagnosed with periodontitis who underwent SRP between 1 January 1 2010 and 31 December 2020 (Figure 1). Data collection involved standardised queries for CDT codes D4341 or D4342. The study protocol was reviewed by the University of Minnesota Institutional Review Board and deemed exempt from human subjects research regulations (STUDY00016576), while also receiving ethical clearance from the BigMouth Consortium. All procedures adhered to the Helsinki Declaration of 1975, as revised in 2013.

### 2.2 Data Extraction and Variable Definition

Patients were categorised into mutually exclusive groups: a control group receiving ‘SRP only’ and test groups receiving ‘SRP + antibiotics’ (amoxicillin, metronidazole, doxycycline, azithromycin, clindamycin, ciprofloxacin or amoxicillin + metronidazole) prescribed within 7 days of the procedure.

Clinical outcomes were assessed by comparing baseline and re‐evaluation measurements:

- Patient level: Changes in mean PD, mean CAL, percentage of sites with PD ≥ 4 mm and percentage of sites with bleeding on probing (BOP). Two composite endpoints were also calculated: ‘Residual burden’ (remaining sites with PD ≥ 4 mm) and ‘clinical success’ (patients with ≤ 10% of sites having PD ≥ 4 mm).
- Tooth level: Categorical changes in tooth mobility and furcation involvement, classified as either ‘worsened’ or ‘not worsened’.

Baseline variables extracted for adjustment included age, sex, smoking status, self‐reported diabetes, disease severity, number of teeth, clinical provider level and the baseline value of the specific periodontal parameter. A detailed description of the data extraction and variable definition is available in the Appendix (Data S1: Supporting Information and methods).

### 2.3 Statistical Analysis

Descriptive statistics characterised the cohort. Continuous patient‐level outcomes were analysed using ANCOVA and multivariable linear regression, while binary outcomes utilised logistic regression to calculate adjusted odds ratios (ORs). All models controlled for baseline disease, demographics and provider type. Tooth‐level stability was assessed via multilevel logistic regression to account for clustering. Comparative effectiveness was evaluated against ‘SRP only’ and through head‐to‐head antibiotic comparisons. Stratified analyses examined disease severity (using a proxy for 2018 staging) and provider experience. Sensitivity analyses tested deep pocket thresholds (PD ≥ 5 mm), and missing data were managed via multiple imputation. Statistical significance was defined as *p* < 0.05. A detailed description of the statistical methodology, including sensitivity analyses and software specifications, is available in the Appendix (Data S1: Supporting Information and methods).

## 3 Results

The cohort selection process is outlined in Figure 1. The study cohort consisted of 3125 patients who underwent SRP. The baseline characteristics of this population are detailed in Table 1. The mean age of the cohort was 58.2 years, with 54.5% being female. A notable proportion of patients were current smokers (22.0%), and the most prevalent self‐reported systemic conditions included high blood pressure (32.8%), arthritis (15.4%) and diabetes mellitus (14.5%). The main medication types used by the cohort were cardiovascular agents (35.4%), analgesics (21.0%) and diuretics (16.4%) (Table 1).

**TABLE 1:** Baseline characteristics of the SRP patient cohort (*N* = 3125).

Characteristic	Category	Total cohort (*N* = 3125)	SRP only (*N* = 2202)	Amoxicillin (*N* = 389)	Metronidazole (*N* = 112)	Amoxicillin + metronadizole (*N* = 213)	Other antibiotics (*N* = 209)
Age	Mean (SD), years	58.2 (12.5)	58.4 (13.6)	57.8 (12.9)	56.9 (13.1)	56.5 (12.5)	57.9 (13.2)
Gender	Female	1703 (54.5%)	1210 (54.9%)	205 (52.7%)	62 (55.4%)	112 (52.6%)	114 (54.5%)
	Male	1422 (45.5%)	992 (45.1%)	184 (47.3%)	50 (44.6%)	101 (47.4%)	95 (45.5%)
Race	White	1288 (41.2%)	908 (41.2%)	160 (41.1%)	46 (41.1%)	88 (41.3%)	86 (41.1%)
	Black or African American	791 (25.3%)	558 (25.3%)	98 (25.2%)	28 (25.0%)	54 (25.4%)	53 (25.4%)
	Hispanic or Latino	494 (15.8%)	348 (15.8%)	61 (15.7%)	18 (16.1%)	34 (16.0%)	33 (15.8%)
	Asian	281 (9.0%)	198 (9.0%)	35 (9.0%)	10 (8.9%)	19 (8.9%)	19 (9.1%)
	Other/not specified	271 (8.7%)	190 (8.7%)	34 (8.7%)	10 (8.9%)	18 (8.5%)	18 (8.6%)
Smoking status	Current smoker	688 (22.0%)	485 (22.0%)	86 (22.1%)	25 (22.3%)	46 (21.6%)	46 (22.0%)
Provider type	DMD/DDS student	2156 (69.0%)	1520 (69.0%)	268 (68.9%)	77 (68.8%)	147 (69.0%)	144 (68.9%)
	Resident	781 (25.0%)	551 (25.0%)	97 (24.9%)	28 (25.0%)	53 (24.9%)	52 (24.9%)
	Faculty	188 (6.0%)	131 (6.0%)	24 (6.2%)	7 (6.3%)	13 (6.1%)	13 (6.2%)
Systemic diseases (prevalence)	High blood pressure	1025 (32.8%)	732 (33.2%)	125 (32.1%)	35 (31.3%)	64 (30.0%)	69 (33.0%)
	Arthritis	481 (15.4%)	339 (15.4%)	60 (15.4%)	17 (15.2%)	33 (15.5%)	32 (15.3%)
	Diabetes mellitus	453 (14.5%)	320 (14.5%)	56 (14.4%)	16 (14.3%)	31 (14.6%)	30 (14.4%)
	Cardiovascular disease	316 (10.1%)	223 (10.1%)	39 (10.0%)	11 (9.8%)	22 (10.3%)	21 (10.0%)
	Anxiety or depression	288 (9.2%)	203 (9.2%)	36 (9.3%)	10 (8.9%)	20 (9.4%)	19 (9.1%)
	Osteoporosis	159 (5.1%)	111 (5.1%)	20 (5.1%)	6 (5.4%)	11 (5.2%)	11 (5.3%)
	Kidney disease	116 (3.7%)	82 (3.7%)	14 (3.6%)	4 (3.6%)	8 (3.8%)	8 (3.8%)
	Malignant cancer	97 (3.1%)	69 (3.1%)	12 (3.1%)	3 (2.7%)	7 (3.3%)	6 (2.9%)
Medications	Cardiovascular agents	1105 (35.4%)	778 (35.4%)	138 (35.5%)	40 (35.7%)	75 (35.2%)	74 (35.4%)
	Analgesics (incl. NSAIDs)	656 (21.0%)	461 (21.0%)	82 (21.1%)	24 (21.4%)	45 (21.1%)	44 (21.1%)
	Diuretics	512 (16.4%)	361 (16.4%)	64 (16.5%)	18 (16.1%)	35 (16.4%)	34 (16.3%)
	Antidepressants/psychotropics	391 (12.5%)	275 (12.5%)	49 (12.6%)	14 (12.5%)	27 (12.7%)	26 (12.4%)
	Antidiabetic agents	359 (11.5%)	254 (11.5%)	45 (11.6%)	13 (11.6%)	24 (11.3%)	24 (11.5%)
	Statins	301 (9.6%)	213 (9.6%)	37 (9.5%)	11 (9.8%)	20 (9.4%)	20 (9.6%)
	Thyroid supplements	188 (6.0%)	131 (6.0%)	23 (5.9%)	7 (6.3%)	13 (6.1%)	13 (6.2%)
	Antibiotics (any reason)	156 (5.0%)	110 (5.0%)	19 (4.9%)	6 (5.4%)	11 (5.2%)	10 (4.8%)
Baseline periodontal status	Number of teeth (mean, SD)	24.1 (4.5)	24.1 (4.5)	24.2 (4.4)	24.0 (4.6)	23.9 (4.5)	24.1 (4.5)
	Mean PD (mm)	3.41 (0.82)	3.38 (0.64)	3.45 (0.66)	3.48 (0.67)	3.68 (0.71)	3.42 (0.65)
	Mean CAL (mm)	3.95 (1.31)	3.91 (0.90)	4.01 (0.93)	4.05 (0.94)	4.35 (0.98)	3.96 (0.91)
	Mean % sites with PD ≥ 4 mm	27.3% (16.8%)	26.5%	28.1%	28.9%	34.2%	27.5%
	Mean % sites with BOP	30.5% (20.9%)	29.8%	31.5%	31.8%	36.1%	30.2%
Highest mobility class	Class 0	1113 (35.6%)	784 (35.6%)	138 (35.5%)	40 (35.7%)	76 (35.7%)	74 (35.4%)
	Class 1	1531 (49.0%)	1079 (49.0%)	191 (49.1%)	55 (49.1%)	104 (48.8%)	102 (48.8%)
	Class 2	406 (13.0%)	285 (13.0%)	51 (13.1%)	15 (13.4%)	28 (13.1%)	27 (12.9%)
	Class 3	75 (2.4%)	53 (2.4%)	9 (2.3%)	3 (2.7%)	5 (2.3%)	5 (2.4%)
Highest furcation grade	Grade 0	1812 (58.0%)	1276 (58.0%)	226 (58.1%)	65 (58.0%)	124 (58.2%)	121 (57.9%)
	Grade 1	860 (27.5%)	606 (27.5%)	107 (27.5%)	31 (27.7%)	59 (27.7%)	57 (27.3%)
	Grade 2	391 (12.5%)	275 (12.5%)	49 (12.6%)	14 (12.5%)	27 (12.7%)	26 (12.4%)
	Grade 3	62 (2.0%)	44 (2.0%)	8 (2.1%)	2 (1.8%)	4 (1.9%)	4 (1.9%)

*Note*: Other Antibiotics include doxycycline, azithromycin, clindamycin, and ciprofloxacin.

Abbreviations: BOP, bleeding on probing; CAL, clinical attachment level; DDS, Doctor of Dental Surgery; DMD, Doctor of Dental Medicine; mm, millimetres; *N*, number of patients; NSAIDs, non‐steroidal anti‐inflammatory drugs; PD, probing depth; SD, standard deviation.

At the initial examination, the cohort presented with established periodontitis, as indicated by a mean PD of 3.41 mm and a mean CAL of 3.95 mm (Table 1). The inflammatory burden was significant, with an average of 30.5% of sites with BOP and 27.3% of sites with a PD ≥ 4 mm. The majority of patients (64.4%) presented with at least one mobile tooth at baseline, and 42.0% had at least one molar with furcation involvement (Table 1). The most common treatment regimen was ‘SRP Only’ (77.1%), followed by SRP with adjunctive amoxicillin (12.4%) and a combination of amoxicillin and metronidazole (6.8%).

Table 2 presents the adjusted mean change in patient‐level periodontal outcomes. Comparisons against the control revealed that the combination amoxicillin + metronidazole was statistically superior to SRP Only for reductions in PD (*p* < 0.001), percentage of sites with PD ≥ 4 mm (*p* = 0.008) and BOP (*p* < 0.001), residual burden (*p* = 0.003) and clinical success (*p* = 0.001). However, results for CAL were mixed. The adjusted mean CAL change was −0.50 mm for the combination group, which was not statistically significantly different from the −0.33 mm observed in the ‘SRP only’ group (*p* = 0.232). In contrast, amoxicillin monotherapy resulted in a CAL change of −0.30 mm (*p* = 0.211). Notably, azithromycin and doxycycline were the only regimens to demonstrate statistically significant improvements in CAL compared to SRP (−1.10 mm, *p* = 0.037 and −0.62 mm, *p* = 0.047, respectively).

**TABLE 2:** Adjusted mean change in patient‐level periodontal outcomes by treatment regimen.

Treatment regimen	*N*	Change in mean PD (mm) [95% CI]	*p*‐value (vs. SRP)	Change in mean CAL (mm) [95% CI]	*p*‐value (vs. SRP)	Change in % sites PD ≥ 4 mm [95% CI]	*p*‐value (vs. SRP)	Change in % sites with BOP [95% CI]	*p*‐value (vs. SRP)	Residual burden (%)	*p*‐value (vs SRP)	Clinical success (%)	*p*‐value (vs. SRP)
SRP only	2202	−0.35 [−0.36, −0.33]	—	−0.33 [−0.35, −0.30]	—	−11.9% [−12.3, −11.4]	—	−14.2%[−15.5, −12.9]	—	14.2%	—	41.5%	—
Amoxicillin	389	−0.33 [−0.35, −0.30]	0.167	−0.30 [−0.34, −0.25]	0.211	−10.9% [−11.7, −10.1]	0.035	−12.8%[−15.1, −10.5]	0.280	13.8%	0.352	44.1%	0.180
Metronidazole	112	−0.32 [−0.54, −0.10]	0.821	−0.24 [−0.46, −0.02]	0.479	−12.5% [−23.0, −1.9]	0.911	−10.5%[−28.1, 7.1]	0.760	13.1%	0.810	50.0%	0.615
Amoxicillin + metronidazol	213	−0.70[−0.87, −0.53]	< 0.001	−0.50 [−0.77, −0.22]	0.232	−16.8%[−20.5, −13.2]	0.008	−25.4%[−36.2, −14.6]	< 0.001	8.9%	0.003	63.5%	0.001
Doxycycline	61	−0.56[−0.73, −0.39]	0.025	−0.62 [−0.89, −0.34]	0.047	−18.1%[−24.0, −12.2]	0.045	−18.5%[−29.8, −7.2]	0.350	10.5%	0.125	57.7%	0.108
Clindamycin	102	−0.31 [−0.38, −0.24]	0.277	−0.38 [−0.52, −0.24]	0.442	−11.8% [−14.3, −9.4]	0.991	−11.9%[−18.5, −5.3]	0.520	14.5%	0.156	40.0%	0.120
Azithromycin	27	−0.83[−1.13, −0.54]	0.012	−1.10 [−1.70, −0.49]	0.037	−21.8%[−28.7, −14.9]	0.020	−31.2%[−55.1, −7.3]	0.045	9.2%	0.880	66.7%	0.825
Ciprofloxacin	19	−0.38 [−0.79, 0.04]	0.895	−0.28 [−0.61, 0.05]	0.798	−11.5% [−23.2, 0.2]	0.953	−13.0%[−45.1, 19.1]	0.920	12.8%	0.670	50.0%	0.750

*Note*: The table shows the adjusted mean improvement for patient‐level metrics, comparing each regimen to the ‘SRP only’ control. All models are adjusted for baseline severity, age, smoking, sex, diabetes, number of teeth and provider type. Residual burden = Percentage of sites with PD ≥ 4 mm remaining at follow‐up. Clinical success = Percentage of patients with ≤ 10% of sites having PD ≥ 4 mm at follow‐up.

Abbreviations: BOP, bleeding on probing; CAL, clinical attachment level; PD, probing depth.

To rigorously evaluate the comparative effectiveness of the antibiotic regimens, direct head‐to‐head comparisons were performed (Table 3). The combination therapy demonstrated statistically superior clinical improvements in PD reduction compared to amoxicillin, metronidazole and clindamycin, but did not show statistically significant differences against doxycycline, azithromycin or ciprofloxacin. Specifically, the combination group achieved a mean PD reduction difference of −0.37 mm compared to amoxicillin (*p* < 0.001) and −0.39 mm compared to clindamycin (*p* < 0.001). Furthermore, the combination regimen proved significantly more effective than metronidazole alone, resulting in a 0.38 mm greater reduction in PD (*p* = 0.017). However, in contrast to PD, the differences in CAL gain were not statistically significant in the comparisons against amoxicillin (*p* = 0.164) or metronidazole (*p* = 0.166). Among the single‐agent comparisons, amoxicillin monotherapy proved significantly less effective than azithromycin, which demonstrated superior results in PD reduction (*p* = 0.010), CAL gain (*p* = 0.032) and BOP reduction (*p* = 0.045).

**TABLE 3:** Mean differences for head‐to‐head comparisons of antibiotic regimens.

Comparison (Group A vs. Group B)	Mean PD change diff. [95% CI] (*p*)	Mean CAL change diff. [95% CI] (*p*)	Change % sites PD ≥ 4 mm diff. [95% CI] (*p*)	Change % BOP diff. [95% CI] (*p*)	Residual burden diff. [95% CI] (*p*)	Clinical success diff. [95% CI] (*p*)
Amoxicillin + metronidazole vs. amoxicillin	−0.37 [−0.49, −0.25] (< 0.001)	−0.20 [−0.35, −0.05] (0.164)	−5.9% [−9.5, −2.3] (0.006)	−12.6% [−18.2, −7.0] (0.004)	−4.9% [−8.3, −1.5] (0.005)	+19.4% [+7.7, +31.1] (0.003)
Amoxicillin + metronidazole vs. metronidazole	−0.38 [−0.68, −0.08] (0.017)	−0.26 [−0.58, 0.06] (0.166)	−4.3% [−10.5, 1.9] (0.210)	−14.9% [−28.5, −1.3] (0.223)	−4.2% [−10.5, 2.1] (0.210)	+13.5% [−20.5, 47.5] (0.290)
Amoxicillin + metronidazole vs. clindamycin	−0.39 [−0.52, −0.26] (< 0.001)	−0.12 [−0.28, 0.04] (0.470)	−5.0% [−8.8, −1.2] (0.015)	−13.5% [−20.5, −6.5] (0.060)	−5.6% [−9.2, −2.0] (0.008)	+23.5% [+10.2, +36.8] (0.010)
Amoxicillin + metronidazole vs. doxycycline	−0.14 [−0.35, 0.07] (0.242)	+0.12 [−0.15, 0.39] (0.537)	+1.3% [−3.5, 6.1] (0.450)	−6.9% [−15.5, 1.7] (0.152)	−1.6% [−5.8, 2.6] (0.450)	+5.8% [−10.5, 22.1] (0.580)
Amoxicillin + metronidazole vs. azithromycin	+0.13 [−0.18, 0.44] (0.458)	+0.60 [−0.05, 1.25] (0.102)	+5.0% [−2.5, 12.5] (0.920)	+5.8% [−8.5, 20.1] (0.524)	−0.3% [−4.5, 3.9] (0.920)	−3.2% [−25.5, 19.1] (0.850)
Amoxicillin + metronidazole vs. ciprofloxacin	−0.32 [−0.75, 0.11] (0.150)	−0.22 [−0.68, 0.24] (0.380)	−5.3% [−15.5, 4.9] (0.220)	−12.4% [−32.5, 7.7] (0.180)	−3.9% [−9.5, 1.7] (0.160)	+13.5% [−28.5, 55.5] (0.310)
Amoxicillin vs. metronidazole	−0.01 [−0.32, 0.30] (0.959)	−0.06 [−0.38, 0.26] (0.644)	+1.6% [−5.5, 8.7] (0.825)	−2.3% [−12.5, 7.9] (0.760)	+0.7% [−4.5, 5.9] (0.825)	−5.9% [−40.5, 28.7] (0.615)
Amoxicillin vs. doxycycline	+0.23 [0.05, 0.41] (0.015)	+0.32 [0.02, 0.62] (0.031)	+7.2% [0.5, 13.9] (0.045)	+5.7% [−5.2, 16.6] (0.350)	+3.3% [−0.5, 7.1] (0.085)	−13.6% [−32.5, 5.3] (0.170)
Amoxicillin vs. azithromycin	+0.50 [0.12, 0.88] (0.010)	+0.80 [0.07, 1.53] (0.032)	+10.9% [2.5, 19.3] (0.020)	+18.4% [0.5, 36.3] (0.045)	+4.6% [−1.2, 10.4] (0.120)	−22.6% [−55.2, 10.0] (0.180)
Amoxicillin vs. clindamycin	−0.02 [−0.12, 0.08] (0.635)	+0.08 [−0.05, 0.21] (0.251)	+0.9% [−1.5, 3.3] (0.991)	−0.9% [−4.2, 2.4] (0.520)	−0.7% [−3.2, 1.8] (0.650)	+4.1% [−7.5, 15.7] (0.680)
Amoxicillin vs. ciprofloxacin	+0.05 [−0.35, 0.45] (0.810)	−0.02 [−0.45, 0.41] (0.930)	+0.6% [−8.5, 9.7] (0.900)	+0.2% [−15.5, 15.9] (0.920)	+1.0% [−4.2, 6.2] (0.720)	−5.9% [−45.5, 33.7] (0.820)
Metronidazole vs. doxycycline	+0.24 [−0.15, 0.63] (0.177)	+0.38 [−0.02, 0.78] (0.052)	+5.6% [−5.5, 16.7] (0.395)	+8.0% [−12.5, 28.5] (0.469)	+2.6% [−2.5, 7.7] (0.578)	−7.7% [−48.5, 33.1] (0.760)
Metronidazole vs. clindamycin	−0.01 [−0.32, 0.30] (0.663)	+0.14 [−0.18, 0.46] (0.320)	−0.7% [−8.5, 7.1] (0.648)	+1.4% [−15.5, 18.3] (0.505)	−1.4% [−5.5, 2.7] (0.232)	+10.0% [−25.5, 45.5] (0.680)
Metronidazole vs. azithromycin	+0.51 [−0.15, 1.17] (0.140)	+0.86 [0.15, 1.57] (0.018)	+9.3% [−12.5, 31.1] (0.505)	+20.7% [−10.5, 51.9] (0.279)	+3.9% [−5.5, 13.3] (0.623)	−16.7% [−65.5, 32.1] (0.420)
Metronidazole vs. ciprofloxacin	+0.06 [−0.55, 0.67] (0.930)	+0.04 [−0.62, 0.70] (0.848)	−1.0% [−25.5, 23.5] (0.360)	+2.5% [−35.5, 40.5] (0.860)	+0.3% [−8.5, 9.1] (0.724)	0.0% [−55.5, 55.5] (0.980)
Doxycycline vs. clindamycin	−0.25 [−0.45, −0.05] (0.015)	−0.24 [−0.48, −0.00] (0.152)	−6.3% [−12.2, −0.4] (0.035)	−6.6% [−15.5, 2.3] (0.906)	−4.0% [−9.5, 1.5] (0.248)	+17.7% [−8.5, 43.9] (0.180)
Doxycycline vs. azithromycin	+0.27 [−0.42, 0.96] (0.608)	+0.48 [−0.25, 1.21] (0.155)	+3.7% [−10.5, 17.9] (0.869)	+12.7% [−15.5, 40.9] (0.392)	+1.3% [−5.5, 8.1] (0.978)	−9.0% [−45.5, 27.5] (0.650)
Doxycycline vs. ciprofloxacin	−0.18 [−0.65, 0.29] (0.496)	−0.34 [−0.85, 0.17] (0.163)	−6.6% [−22.5, 9.3] (0.513)	−5.5% [−32.5, 21.5] (0.708)	−2.3% [−9.5, 4.9] (0.571)	+7.7% [−38.5, 53.9] (0.740)
Clindamycin vs. azithromycin	+0.52 [−0.02, 1.06] (0.051)	+0.72 [0.05, 1.39] (0.037)	+10.0% [−2.5, 22.5] (0.137)	+19.3% [−5.5, 44.1] (0.384)	+5.3% [−2.5, 13.1] (0.382)	−26.7% [−58.5, 5.1] (0.150)
Clindamycin vs. ciprofloxacin	+0.07 [−0.42, 0.56] (0.758)	−0.10 [−0.65, 0.45] (0.608)	−0.3% [−12.5, 11.9] (0.293)	+1.1% [−25.5, 27.7] (0.737)	+1.7% [−5.5, 8.9] (0.440)	−10.0% [−52.5, 32.5] (0.660)
Azithromycin vs. ciprofloxacin	−0.45 [−1.15, 0.25] (0.350)	−0.82 [−1.55, −0.09] (0.030)	−10.3% [−32.5, 11.9] (0.487)	−18.2% [−55.5, 19.1] (0.340)	−3.6% [−12.5, 5.3] (0.580)	+16.7% [−45.5, 78.9] (0.490)

*Note*: This table shows the results of the direct comparison between the combination therapy and other regimens. *p*‐values are adjusted for multiple comparisons. Residual burden = Percentage of sites with PD ≥ 4 mm remaining at follow‐up. Clinical success = Percentage of patients with ≤ 10% of sites having PD ≥ 4 mm at follow‐up.

Abbreviations: BOP, bleeding on probing; CAL, clinical attachment level; PD, probing depth.

To identify which patient profiles benefit most from adjunctive therapy, the analysis was stratified by baseline disease severity (Table 4). The interaction between disease severity and treatment response was assessed for all antibiotic regimens. The amoxicillin + metronidazole combination provided the most substantial additional benefit in patients with severe periodontitis, yielding a significantly greater CAL gain of 0.35 mm (*p* < 0.001) compared to SRP alone. In contrast, for patients with mild disease, the additional benefit of the combination therapy regarding CAL was smaller (0.15 mm) and did not reach statistical significance (*p* = 0.068). A similar trend was observed for PD reduction, where the magnitude of the benefit was consistently higher in the severe disease cohort (−0.55 mm, *p* < 0.001) compared to the mild cohort (−0.20 mm, *p* < 0.01). In severe cases, the combination therapy demonstrated a profound advantage over SRP alone, achieving an OR for clinical success of 4.55 (*p* < 0.001) and reducing the residual burden of diseased sites by 11.4% (*p* < 0.001). In the mild‐to‐moderate stratum (Stage I/II), adjunctive antibiotics generally showed limited efficacy over SRP alone. However, the amoxicillin + metronidazole combination achieved a statistically significant reduction in residual disease burden compared to SRP (mean difference: −3.8%; 95% CI: −6.8 to −0.8; *p* = 0.030). No other antibiotic regimen provided a significant benefit for this outcome in the mild‐to‐moderate group.

**TABLE 4:** Comprehensive head‐to‐head comparisons stratified by disease stage.

Comparison (Group A vs. Group B)	Mean PD change diff. [95% CI] (*p*) mild/severe	Mean CAL change diff. [95% CI] (*p*) mild/severe	Change % sites PD ≥ 4 mm diff. [95% CI] (*p*) mild/severe	Change % BOP diff. [95% CI] (*p*) mild/severe	Residual burden diff. [95% CI] (*p*) mild/severe	Clinical success OR [95% CI] (*p*) mild/severe
Amoxicillin + metronidazole vs. SRP only	−0.20 [−0.30, −0.10] (< 0.01)/−0.55 [−0.65, −0.45] (< 0.001)	−0.15 [−0.31, 0.01] (0.068)/−0.35 [−0.48, −0.22] (< 0.001)	−4.5% [−8.2, −0.8] (0.020)/−12.5% [−16.5, −8.5] (< 0.001)	−6.2% [−10.8, −1.6] (0.010)/−18.4% [−23.5, −13.3] (< 0.001)	−3.8% [−6.8, −0.8] (0.030)/−11.4% [−14.5, −8.3] (< 0.001)	1.62 [0.65, 4.02] (0.380)/4.55 [2.35, 8.80] (< 0.001)
Amoxicillin + metronidazole vs. amoxicillin	−0.12 [−0.25, 0.01] (0.085)/−0.30 [−0.42, −0.18] (< 0.001)	−0.10 [−0.22, 0.02] (0.120)/−0.15 [−0.28, −0.02] (0.015)	−2.8% [−6.5, 0.9] (0.150)/−8.4% [−13.2, −3.6] (0.004)	−3.5% [−7.8, 0.8] (0.120)/−12.6% [−17.2, −8.0] (< 0.001)	−2.5% [−5.8, 0.8] (0.150)/−8.2% [−11.8, −4.6] (0.003)	1.50 [0.58, 3.85] (0.350)/3.20 [1.65, 6.20] (< 0.001)
Amoxicillin + metronidazole vs. metronidazole	−0.10 [−0.35, 0.15] (0.150)/−0.25 [−0.48, −0.02] (0.035)	−0.07 [−0.32, 0.18] (0.250)/−0.13 [−0.38, 0.12] (0.150)	−2.2% [−8.5, 4.1] (0.220)/−6.0% [−12.5, 0.5] (0.060)	−2.8% [−8.5, 2.9] (0.250)/−10.5% [−16.8, −4.2] (0.045)	−2.0% [−6.2, 2.2] (0.280)/−5.8% [−10.5, −1.1] (0.040)	1.75 [0.35, 8.75] (0.450)/1.72 [0.55, 5.38] (0.450)
Amoxicillin + metronidazole vs. clindamycin	−0.15 [−0.28, −0.02] (0.045)/−0.40 [−0.55, −0.25] (< 0.001)	−0.13 [−0.28, 0.02] (0.080)/−0.25 [−0.42, −0.08] (0.005)	−3.5% [−7.2, 0.2] (0.045)/−9.5% [−15.2, −3.8] (< 0.001)	−4.8% [−9.5, −0.1] (0.040)/−14.2% [−19.8, −8.6] (< 0.001)	−3.0% [−5.8, −0.2] (0.048)/−8.5% [−12.8, −4.2] (0.002)	1.52 [0.52, 4.45] (0.320)/5.18 [2.10, 12.75] (0.002)
Amoxicillin + metronidazole vs. doxycycline	−0.15 [−0.35, 0.05] (0.045)/−0.20 [−0.45, 0.05] (0.085)	−0.13 [−0.38, 0.12] (0.090)/−0.10 [−0.35, 0.15] (0.250)	−3.3% [−6.5, 0.1] (0.045)/−4.5% [−10.2, 1.2] (0.150)	−4.7% [−9.2, −0.2] (0.040)/−6.8% [−12.5, −1.1] (0.120)	−2.8% [−6.5, 0.9] (0.065)/−4.2% [−9.5, 1.1] (0.180)	1.45 [0.45, 4.65] (0.650)/1.15 [0.35, 3.75] (0.820)
Amoxicillin + metronidazole vs. azithromycin	−0.15 [−0.42, 0.12] (0.045)/−0.10 [−0.38, 0.18] (0.350)	−0.13 [−0.45, 0.19] (0.120)/−0.05 [−0.35, 0.25] (0.650)	−3.3% [−6.5, 0.1] (0.045)/−2.5% [−8.5, 3.5] (0.450)	−4.7% [−9.2, −0.2] (0.040)/−3.5% [−9.5, 2.5] (0.350)	−2.8% [−6.5, 0.9] (0.065)/−2.0% [−7.8, 3.8] (0.550)	1.35 [0.32, 5.68] (0.750)/0.88 [0.22, 3.52] (0.850)
Amoxicillin + metronidazole vs. ciprofloxacin	−0.15 [−0.45, 0.15] (0.048)/−0.35 [−0.62, −0.08] (0.015)	−0.13 [−0.48, 0.22] (0.110)/−0.20 [−0.45, 0.05] (0.045)	−3.3% [−6.8, 0.2] (0.048)/−8.0% [−14.5, −1.5] (0.035)	−4.7% [−9.5, 0.1] (0.045)/−11.5% [−18.5, −4.5] (0.020)	−2.8% [−6.2, 0.6] (0.060)/−6.5% [−11.8, −1.2] (0.040)	1.48 [0.35, 6.25] (0.720)/1.70 [0.45, 6.42] (0.310)
Amoxicillin vs. metronidazole	+0.02 [−0.22, 0.26] (0.750)/+0.05 [−0.18, 0.28] (0.650)	+0.03 [−0.21, 0.27] (0.820)/+0.02 [−0.22, 0.26] (0.850)	+0.5% [−5.5, 6.5] (0.850)/+1.5% [−4.5, 7.5] (0.750)	+0.8% [−5.2, 6.8] (0.780)/+2.0% [−3.8, 7.8] (0.650)	+0.4% [−5.2, 6.0] (0.880)/+1.2% [−3.5, 5.9] (0.720)	1.15 [0.25, 5.25] (0.850)/0.54 [0.22, 1.35] (0.450)
Amoxicillin vs. doxycycline	−0.03 [−0.18, 0.12] (0.680)/+0.10 [−0.12, 0.32] (0.350)	−0.03 [−0.18, 0.12] (0.720)/+0.05 [−0.15, 0.25] (0.450)	−0.5% [−3.5, 2.5] (0.750)/+2.5% [−1.5, 6.5] (0.420)	−0.8% [−3.8, 2.2] (0.650)/+3.5% [−1.2, 8.2] (0.350)	−0.5% [−3.2, 2.2] (0.820)/+2.2% [−1.5, 5.9] (0.450)	0.95 [0.32, 2.85] (0.950)/0.36 [0.12, 1.05] (0.120)
Amoxicillin vs. azithromycin	−0.03 [−0.22, 0.16] (0.720)/+0.20 [−0.05, 0.45] (0.150)	−0.03 [−0.25, 0.19] (0.750)/+0.10 [−0.15, 0.35] (0.250)	−0.5% [−4.5, 3.5] (0.820)/+4.5% [−0.5, 9.5] (0.180)	−0.7% [−4.5, 3.1] (0.750)/+6.5% [0.5, 12.5] (0.120)	−0.4% [−3.8, 3.0] (0.850)/+3.5% [−1.2, 8.2] (0.250)	0.88 [0.25, 3.12] (0.850)/0.28 [0.08, 0.95] (0.150)
Amoxicillin vs. clindamycin	−0.03 [−0.15, 0.09] (0.650)/−0.10 [−0.28, 0.08] (0.320)	−0.03 [−0.16, 0.10] (0.720)/−0.05 [−0.22, 0.12] (0.650)	−0.6% [−3.8, 2.6] (0.720)/−2.5% [−7.5, 2.5] (0.450)	−0.8% [−4.2, 2.6] (0.680)/−3.5% [−8.5, 1.5] (0.350)	−0.5% [−3.5, 2.5] (0.780)/−2.2% [−6.5, 2.1] (0.480)	1.02 [0.45, 2.30] (0.980)/1.62 [0.65, 4.05] (0.680)
Amoxicillin vs. ciprofloxacin	−0.03 [−0.25, 0.19] (0.680)/−0.05 [−0.30, 0.20] (0.550)	−0.03 [−0.28, 0.22] (0.750)/−0.05 [−0.28, 0.18] (0.620)	−0.6% [−4.5, 3.3] (0.750)/−1.2% [−5.5, 3.1] (0.720)	−0.8% [−4.8, 3.2] (0.720)/−1.5% [−6.5, 3.5] (0.680)	−0.5% [−4.2, 3.2] (0.820)/−1.0% [−5.2, 3.2] (0.750)	0.98 [0.25, 3.85] (0.650)/0.52 [0.15, 1.85] (0.820)
Amoxicillin vs. SRP only	−0.08 [−0.15, −0.01] (0.045)/−0.25 [−0.32, −0.18] (< 0.01)	−0.05 [−0.12, 0.02] (0.120)/−0.20 [−0.28, −0.12] (< 0.01)	−1.7% [−3.5, 0.1] (0.150)/−6.5% [−9.2, −3.8] (0.004)	−2.5% [−5.2, 0.2] (0.120)/−8.5% [−12.2, −4.8] (0.002)	−1.5% [−3.8, 0.8] (0.220)/−5.5% [−8.5, −2.5] (0.015)	1.08 [0.72, 1.62] (0.850)/1.42 [1.02, 1.98] (0.002)
Metronidazole vs. SRP only	−0.10 [−0.20, 0.00] (0.055)/−0.30 [−0.40, −0.20] (< 0.01)	−0.08 [−0.18, 0.02] (0.095)/−0.22 [−0.35, −0.09] (0.025)	−2.2% [−5.5, 1.1] (0.120)/−7.5% [−11.5, −3.5] (0.008)	−3.2% [−6.5, 0.1] (0.090)/−9.5% [−14.5, −4.5] (0.005)	−1.8% [−4.5, 0.9] (0.180)/−6.2% [−10.2, −2.2] (0.020)	1.25 [0.55, 2.85] (0.550)/2.58 [1.32, 5.05] (0.015)
Ciprofloxacin vs. SRP only	−0.05 [−0.25, 0.15] (0.180)/−0.20 [−0.45, 0.05] (0.120)	−0.02 [−0.22, 0.18] (0.450)/−0.15 [−0.35, 0.05] (0.250)	−1.1% [−4.5, 2.3] (0.280)/−4.5% [−9.5, 0.5] (0.180)	−1.6% [−5.5, 2.3] (0.250)/−6.9% [−12.5, −1.3] (0.065)	−1.0% [−3.8, 1.8] (0.350)/−4.9% [−9.5, −0.3] (0.120)	1.10 [0.35, 3.45] (0.850)/2.68 [0.95, 7.55] (0.060)
Clindamycin vs. SRP only	−0.05 [−0.12, 0.02] (0.150)/−0.15 [−0.25, −0.05] (0.080)	−0.02 [−0.10, 0.06] (0.450)/−0.10 [−0.22, 0.02] (0.250)	−1.0% [−3.5, 1.5] (0.280)/−3.0% [−7.5, 1.5] (0.180)	−1.4% [−4.5, 1.7] (0.250)/−4.2% [−9.5, 1.1] (0.120)	−0.8% [−3.2, 1.6] (0.350)/−2.9% [−7.5, 1.7] (0.180)	1.06 [0.55, 2.05] (0.850)/0.88 [0.45, 1.72] (0.680)
Metronidazole vs. doxycycline	−0.05 [−0.25, 0.15] (0.550)/+0.05 [−0.18, 0.28] (0.650)	−0.06 [−0.25, 0.13] (0.520)/+0.03 [−0.18, 0.24] (0.750)	−1.0% [−4.5, 2.5] (0.580)/+1.0% [−3.5, 5.5] (0.820)	−1.7% [−5.5, 2.1] (0.550)/+1.5% [−3.8, 6.8] (0.780)	−0.8% [−4.2, 2.6] (0.650)/+1.0% [−3.2, 5.2] (0.850)	0.82 [0.22, 3.05] (0.750)/0.65 [0.18, 2.35] (0.650)
Metronidazole vs. clindamycin	−0.05 [−0.22, 0.12] (0.620)/−0.15 [−0.35, 0.05] (0.320)	−0.06 [−0.22, 0.10] (0.650)/−0.10 [−0.32, 0.12] (0.450)	−1.0% [−4.5, 2.5] (0.650)/−3.5% [−8.5, 1.5] (0.350)	−1.7% [−5.2, 1.8] (0.620)/−4.5% [−9.5, 0.5] (0.320)	−0.8% [−4.5, 2.9] (0.680)/−3.0% [−7.5, 1.5] (0.450)	0.88 [0.25, 3.10] (0.720)/3.00 [0.85, 10.5] (0.550)
Metronidazole vs. azithromycin	−0.05 [−0.28, 0.18] (0.650)/+0.15 [−0.12, 0.42] (0.250)	−0.06 [−0.28, 0.16] (0.680)/+0.08 [−0.15, 0.31] (0.450)	−1.0% [−5.5, 3.5] (0.680)/+3.5% [−1.5, 8.5] (0.350)	−1.7% [−6.2, 2.8] (0.650)/+5.5% [−0.5, 11.5] (0.280)	−0.8% [−4.8, 3.2] (0.720)/+2.5% [−2.5, 7.5] (0.450)	0.75 [0.18, 3.15] (0.450)/0.50 [0.12, 2.10] (0.350)
Metronidazole vs. ciprofloxacin	−0.05 [−0.32, 0.22] (0.720)/−0.10 [−0.35, 0.15] (0.450)	−0.06 [−0.32, 0.20] (0.750)/−0.05 [−0.28, 0.18] (0.650)	−1.0% [−5.5, 3.5] (0.750)/−2.5% [−7.5, 2.5] (0.450)	−1.7% [−6.5, 3.1] (0.720)/−3.5% [−8.5, 1.5] (0.420)	−0.8% [−5.2, 3.6] (0.780)/−2.0% [−6.5, 2.5] (0.550)	0.85 [0.15, 4.85] (0.350)/1.00 [0.18, 5.50] (0.980)
Doxycycline vs. clindamycin	0.00 [−0.18, 0.18] (1.000)/−0.20 [−0.38, −0.02] (0.025)	0.00 [−0.18, 0.18] (1.000)/−0.15 [−0.35, 0.05] (0.152)	0.0% [−4.5, 4.5] (1.000)/−4.5% [−9.2, −0.2] (0.045)	0.0% [−5.2, 5.2] (1.000)/−6.5% [−12.5, −0.5] (0.090)	0.0% [−4.5, 4.5] (1.000)/−4.2% [−9.5, 1.1] (0.180)	1.05 [0.35, 3.15] (0.920)/4.50 [1.20, 16.8] (0.090)
Doxycycline vs. azithromycin	0.00 [−0.25, 0.25] (1.000)/+0.10 [−0.18, 0.38] (0.608)	0.00 [−0.25, 0.25] (1.000)/+0.05 [−0.22, 0.32] (0.850)	0.0% [−5.5, 5.5] (1.000)/+2.5% [−2.5, 7.5] (0.650)	0.0% [−6.2, 6.2] (1.000)/+3.5% [−2.5, 9.5] (0.550)	0.0% [−5.2, 5.2] (1.000)/+1.5% [−3.5, 6.5] (0.650)	0.92 [0.22, 3.85] (0.650)/0.78 [0.15, 3.95] (0.650)
Doxycycline vs. ciprofloxacin	0.00 [−0.28, 0.28] (1.000)/−0.15 [−0.45, 0.15] (0.496)	0.00 [−0.28, 0.28] (1.000)/−0.10 [−0.35, 0.15] (0.550)	0.0% [−6.5, 6.5] (1.000)/−3.5% [−9.5, 2.5] (0.450)	0.0% [−7.2, 7.2] (1.000)/−4.5% [−10.5, 1.5] (0.420)	0.0% [−5.8, 5.8] (1.000)/−2.5% [−7.5, 2.5] (0.480)	1.05 [0.22, 5.05] (0.650)/1.50 [0.32, 6.95] (0.740)
Clindamycin vs. azithromycin	0.00 [−0.22, 0.22] (1.000)/+0.30 [0.02, 0.58] (0.051)	0.00 [−0.22, 0.22] (1.000)/+0.20 [0.01, 0.39] (0.037)	0.0% [−5.2, 5.2] (1.000)/+6.5% [0.5, 12.5] (0.137)	0.0% [−6.5, 6.5] (1.000)/+10.5% [2.5, 18.5] (0.090)	0.0% [−4.8, 4.8] (1.000)/+5.5% [0.5, 10.5] (0.182)	0.88 [0.25, 3.10] (0.550)/0.18 [0.05, 0.65] (0.150)
Clindamycin vs. ciprofloxacin	0.00 [−0.28, 0.28] (1.000)/+0.05 [−0.22, 0.32] (0.758)	0.00 [−0.28, 0.28] (1.000)/+0.05 [−0.18, 0.28] (0.820)	0.0% [−6.5, 6.5] (1.000)/+1.0% [−4.5, 6.5] (0.850)	0.0% [−7.2, 7.2] (1.000)/+1.5% [−5.5, 8.5] (0.780)	0.0% [−5.8, 5.8] (1.000)/+1.0% [−4.5, 6.5] (0.850)	0.95 [0.25, 3.65] (0.650)/0.32 [0.08, 1.35] (0.660)
Azithromycin vs. ciprofloxacin	0.00 [−0.35, 0.35] (1.000)/−0.25 [−0.58, 0.08] (0.350)	0.00 [−0.35, 0.35] (1.000)/−0.15 [−0.42, 0.12] (0.450)	0.0% [−8.5, 8.5] (1.000)/−5.5% [−12.5, 1.5] (0.350)	0.0% [−9.5, 9.5] (1.000)/−7.5% [−15.5, 0.5] (0.280)	0.0% [−7.5, 7.5] (1.000)/−4.5% [−10.5, 1.5] (0.350)	1.10 [0.18, 6.75] (1.000)/1.85 [0.35, 9.75] (0.490)
Azithromycin vs. SRP only	−0.05 [−0.18, 0.08] (0.150)/−0.45 [−0.65, −0.25] (0.035)	−0.02 [−0.15, 0.11] (0.450)/−0.30 [−0.55, −0.05] (0.045)	−1.2% [−4.5, 2.1] (0.250)/−10.0% [−16.2, −3.8] (0.040)	−1.5% [−4.8, 1.8] (0.220)/−14.5% [−20.5, −8.5] (0.080)	−1.0% [−3.8, 1.8] (0.350)/−8.0% [−14.5, −1.5] (0.045)	1.25 [0.45, 3.45] (0.650)/3.95 [1.45, 10.75] (0.120)
Doxycycline vs. SRP only	−0.05 [−0.15, 0.05] (0.150)/−0.35 [−0.52, −0.18] (0.015)	−0.02 [−0.12, 0.08] (0.450)/−0.25 [−0.42, −0.08] (0.020)	−1.2% [−3.5, 1.1] (0.250)/−8.0% [−13.5, −2.5] (0.080)	−1.5% [−4.2, 1.2] (0.220)/−11.6% [−17.2, −6.0] (0.150)	−1.0% [−3.2, 1.2] (0.350)/−7.2% [−12.5, −1.9] (0.125)	1.10 [0.45, 2.65] (0.920)/3.10 [1.25, 7.65] (0.010)

*Note*: Residual burden = Percentage of sites with PD ≥ 4 mm remaining at follow‐up. Clinical success = Percentage of patients with ≤ 10% of sites having PD ≥ 4 mm at follow‐up.

Abbreviations: BOP, bleeding on probing; CAL, clinical attachment level; CI, confidence interval; PD, probing depth.

Furthermore, when analysing severe disease specifically, the combination therapy consistently outperformed single‐agent regimens that showed little efficacy in the aggregate analysis; for instance, against amoxicillin alone, the combination achieved a 12.6% greater reduction in BOP (*p* < 0.001) and a 0.30 mm greater PD reduction (*p* < 0.001). Similarly, the combination was superior to ciprofloxacin in the severe cohort, showing a statistically significant advantage in PD reduction (−0.35 mm, *p* = 0.015) and residual burden reduction (−6.5%, *p* = 0.040), differences that were not observed in the mild cohort.

Regarding tooth‐level stability (Table 5), the amoxicillin + metronidazole combination was associated with the numerically lowest proportion of teeth exhibiting worsening mobility (0.9%) and worsening furcation involvement (0.7%). The amoxicillin + metronidazole combination demonstrated the most robust protective effect, significantly reducing the odds of worsening mobility by 63% (aOR 0.37, *p* < 0.001) and worsening furcation status by 50% (aOR 0.50, *p* = 0.012) compared to SRP alone. Among the monotherapies, metronidazole was the only agent to achieve statistical significance for both outcomes, lowering the odds of worsening mobility (*p* = 0.008) and furcation involvement (*p* = 0.048) relative to the control. Amoxicillin also provided significant stability regarding tooth mobility (*p* = 0.002) but failed to reach statistical significance for furcation changes (*p* = 0.086). Notably, doxycycline, clindamycin, azithromycin and ciprofloxacin did not demonstrate any statistically significant benefit over SRP alone in preventing the deterioration of either mobility or furcation status (*p* > 0.05 for all).

**TABLE 5:** Tooth‐level outcomes—Proportion of teeth worsening in mobility or furcation status.

Comparison (Group A vs. Group B)	% Worsening mobility (Group A/Group B)	Worsening mobility aOR [95% CI] (*p*‐value)	% Worsening furcation (Group A/Group B)	Worsening furcation aOR [95% CI] (*p*‐value)
Amoxicillin + metronidazole vs. SRP only	0.9%/2.4%	0.37 [0.22, 0.62] (*p* < 0.001)	0.7% /1.4%	0.50 [0.29, 0.86] (*p* = 0.012)
Amoxicillin + metronidazole vs. amoxicillin	0.9%/1.4%	0.64 [0.35, 1.15] (*p* = 0.166)	0.7%/1.0%	0.70 [0.38, 1.30] (*p* = 0.280)
Amoxicillin + metronidazole vs. metronidazole	0.9%/1.1%	0.82 [0.38, 1.76] (*p* = 0.750)	0.7%/0.7%	1.00 [0.45, 2.22] (*p* = 1.000)
Amox + metro vs. clindamycin	0.9%/1.5%	0.60 [0.28, 1.28] (*p* = 0.150)	0.7%/1.0%	0.70 [0.30, 1.63] (*p* = 0.320)
Amoxicillin + metronidazole vs. doxycycline	0.9%/1.5%	0.60 [0.28, 1.28] (*p* = 0.180)	0.7%/1.0%	0.70 [0.30, 1.63] (*p* = 0.420)
Amoxicillin + metronidazole vs. azithromycin	0.9%/1.5%	0.60 [0.25, 1.45] (*p* = 0.220)	0.7%/1.0%	0.70 [0.25, 1.95] (*p* = 0.450)
Amoxicillin + metronidazole vs. ciprofloxacin	0.9%/1.5%	0.60 [0.22, 1.65] (*p* = 0.250)	0.7%/1.0%	0.70 [0.22, 2.20] (*p* = 0.480)
Amoxicillin vs. metronidazole	1.4%/1.1%	1.29 [0.65, 2.55] (*p* = 0.650)	1.0%/0.7%	1.42 [0.68, 2.95] (*p* = 0.720)
Amoxicillin vs. doxycycline	1.4%/1.5%	0.93 [0.55, 1.58] (*p* = 0.850)	1.0%/1.0%	1.00 [0.52, 1.92] (*p* = 1.000)
Amoxicillin vs. azithromycin	1.4%/1.5%	0.93 [0.45, 1.95] (*p* = 0.880)	1.0%/1.0%	1.00 [0.42, 2.40] (*p* = 1.000)
Amoxicillin vs. clindamycin	1.4%/1.5%	0.93 [0.52, 1.66] (*p* = 0.750)	1.0%/1.0%	1.00 [0.50, 2.00] (*p* = 1.000)
Amoxicillin vs. ciprofloxacin	1.4%/1.5%	0.93 [0.38, 2.28] (*p* = 0.820)	1.0%/1.0%	1.00 [0.35, 2.85] (*p* = 1.000)
Amoxicillin vs. SRP only	1.4%/2.4%	0.58 [0.41, 0.82] (*p* = 0.002)	1.0%/1.4%	0.71 [0.48, 1.05] (*p* = 0.086)
Metronidazole vs. doxycycline	1.1%/1.5%	0.73 [0.32, 1.68] (*p* = 0.550)	0.7%/1.0%	0.70 [0.28, 1.75] (*p* = 0.650)
Metronidazole vs. clindamycin	1.1%/1.5%	0.73 [0.32, 1.68] (*p* = 0.480)	0.7%/1.0%	0.70 [0.28, 1.75] (*p* = 0.580)
Metronidazole vs. azithromycin	1.1%/1.5%	0.73 [0.28, 1.92] (*p* = 0.520)	0.7%/1.0%	0.70 [0.25, 1.95] (*p* = 0.620)
Metronidazole vs. ciprofloxacin	1.1%/1.5%	0.73 [0.25, 2.15] (*p* = 0.550)	0.7%/1.0%	0.70 [0.22, 2.20] (*p* = 0.650)
Metronidazole vs. SRP only	1.1%/2.4%	0.45 [0.25, 0.81] (*p* = 0.008)	0.7%/1.4%	0.50 [0.25, 0.99] (*p* = 0.048)
Doxycycline vs. clindamycin	1.5%/1.5%	1.00 [0.45, 2.22] (*p* = 1.000)	1.0%/1.0%	1.00 [0.42, 2.38] (*p* = 1.000)
Doxycycline vs. azithromycin	1.5%/1.5%	1.00 [0.38, 2.65] (*p* = 1.000)	1.0%/1.0%	1.00 [0.35, 2.85] (*p* = 1.000)
Doxycycline vs. ciprofloxacin	1.5%/1.5%	1.00 [0.32, 3.12] (*p* = 1.000)	1.0%/1.0%	1.00 [0.28, 3.55] (*p* = 1.000)
Doxycycline vs. SRP only	1.5%/2.4%	0.63 [0.38, 1.05] (*p* = 0.120)	1.0%/1.4%	0.71 [0.40, 1.25] (*p* = 0.210)
Clindamycin vs. azithromycin	1.5%/1.5%	1.00 [0.38, 2.65] (*p* = 1.000)	1.0%/1.0%	1.00 [0.35, 2.85] (*p* = 1.000)
Clindamycin vs. ciprofloxacin	1.5%/1.5%	1.00 [0.32, 3.12] (*p* = 1.000)	1.0%/1.0%	1.00 [0.28, 3.55] (*p* = 1.000)
Clindamycin vs. SRP only	1.5%/2.4%	0.63 [0.38, 1.05] (*p* = 0.090)	1.0%/1.4%	0.71 [0.40, 1.25] (*p* = 0.180)
Azithromycin vs. ciprofloxacin	1.5%/1.5%	1.00 [0.25, 4.00] (*p* = 1.000)	1.0%/1.0%	1.00 [0.22, 4.50] (*p* = 1.000)
Azithromycin vs. SRP only	1.5%/2.4%	0.63 [0.28, 1.42] (*p* = 0.150)	1.0%/1.4%	0.71 [0.28, 1.80] (*p* = 0.250)
Ciprofloxacin vs. SRP only	1.5%/2.4%	0.63 [0.22, 1.80] (*p* = 0.250)	1.0%/1.4%	0.71 [0.22, 2.30] (*p* = 0.350)

*Note*: This table presents the percentage of teeth exhibiting worsening mobility or furcation status following therapy, along with the aOR and 95% CIs.

Abbreviations: aOR, adjusted odds ratio; CI, confidence interval; Ref, reference group.

The adjusted mean changes in periodontal outcomes stratified by provider level are presented in Table SS1. This analysis details the specific clinical improvements achieved by DMD/DDS students, residents and faculty, accounting for the potential influence of operator experience on the therapeutic outcomes.

## 4 Discussion

This large‐scale, retrospective cohort study provides real‐world evidence on the comparative effectiveness of adjunctive systemic antibiotics in the non‐surgical treatment of periodontitis. The primary finding of this analysis is that the use of systemic antibiotics in conjunction with SRP leads to statistically significant and clinically meaningful improvements across a comprehensive range of periodontal outcomes compared to SRP alone. Consistent with the study hypothesis, the combination of amoxicillin and metronidazole emerged as the most robust regimen, demonstrating superiority not only over SRP alone but also over several monotherapies in head‐to‐head comparisons.

### 4.1 Impact of Disease Severity

Perhaps the most critical finding of this study is the differential impact of antibiotics based on disease severity. Our stratified analysis reveals that the benefits of the amoxicillin + metronidazole combination are largely driven by patients with severe periodontitis (Stage III/IV proxy). In this high‐severity cohort, the combination therapy provided a massive additional benefit, achieving a clinical success OR of 4.55 (*p* < 0.001) and reducing the residual burden of diseased sites by 11.4% compared to SRP alone. While we observed a statistically significant reduction in residual burden for Stage I/II patients treated with amoxicillin + metronidazole (*p* = 0.030), the magnitude of this effect (−3.8%) was considerably smaller than that observed in Stage III/IV patients (−11.4%). This strongly supports the concept of targeted antibiotic stewardship; the indiscriminate use of this potent combination in mild cases offers negligible clinical gain while contributing to antibiotic resistance, whereas its application in severe cases is clinically justified and highly effective.

### 4.2 Comparative Effectiveness of Regimens

While systematic reviews have established the efficacy of amoxicillin plus metronidazole (Sgolastra et al. 2021; Zandbergen et al. 2016), our head‐to‐head comparisons clarify its standing against other options. The combination therapy was statistically superior to amoxicillin monotherapy, metronidazole monotherapy and clindamycin regarding PD reduction. Interestingly, while the combination resulted in the greatest overall reduction in inflammatory burden (BOP) and deep pockets, azithromycin and doxycycline also showed statistically significant gains in CAL compared to SRP alone. Specifically, azithromycin performed surprisingly well in direct comparisons against amoxicillin monotherapy. However, the sample sizes for these alternatives were smaller than the main cohort, and the combination regimen remained the only therapy to consistently improve all evaluated patient‐level metrics including PD, BOP and clinical success.

### 4.3 Tooth‐Level Stability

Beyond average patient‐level improvements, this study uniquely evaluated the ‘survival’ of compromised teeth. The amoxicillin and metronidazole combination demonstrated a protective effect, significantly reducing the odds of worsening mobility by 63% and worsening furcation status by 50% compared to SRP alone. This suggests that aggressive chemical biofilm control can stabilise the attachment apparatus of teeth with complex anatomy or compromised support. Notably, metronidazole monotherapy also provided significant stability benefits for both mobility and furcation, whereas other agents like clindamycin and doxycycline did not. This finding provides clinicians with evidence that specific antibiotic regimens can help prevent the deterioration of ‘hopeless’ or questionable teeth, potentially altering the prognosis.

### 4.4 Context and Literature

The magnitude of improvement observed with the combination therapy (an additional ~0.37 mm PD reduction and ~0.30 mm CAL gain vs. SRP) aligns with data from RCTs (Herrera et al. 2002; Haffajee et al. 2003). However, by analysing a diverse, real‐world population, we demonstrate that these results are translatable to routine practice, provided the patient selection is appropriate. Our results contradict the notion of a ‘one‐size‐fits‐all’ efficacy; for example, while amoxicillin monotherapy is frequently prescribed, our data indicate it was significantly less effective than the combination therapy and azithromycin in reducing deep pockets, questioning its utility as a stand‐alone adjunct.

### 4.5 Strengths and Limitations

A key strength of this research is the use of the BigMouth Consortium database, which allowed for a large sample size (*N* = 3125), and the statistical power to adjust for critical covariates. By using multivariable regression models, the analysis adjusted for baseline disease severity, age, smoking status, sex, diabetes and provider level, thereby isolating the independent effect of the therapeutic regimens. Unlike RCTs with strict inclusion criteria, this cohort reflects the heterogeneity of actual clinical practice.

The study's retrospective nature carries inherent limitations. The data was collected during routine care, not under a standardised research protocol, which may introduce variability. The relatively short follow‐up period between initial therapy and re‐evaluation means our results reflect initial healing responses and not necessarily long‐term periodontal stability. We were unable to control for factors such as patient compliance with the prescribed medication, specific oral hygiene practices or the rationale behind a clinician's decision to prescribe an antibiotic. Furthermore, the EHRs utilised a broad diagnostic code for ‘periodontitis’ that was not sub‐classified by stage or grade, which are known to influence treatment response (Kapoor et al. 2012; Slots 2002). To overcome this limitation, we applied a post hoc operational definition based on clinical attachment loss to serve as a proxy for disease severity in our stratified analysis. Furthermore, a limitation inherent to the retrospective design is that specific details of the antibiotic prescriptions—including dosage, dosing frequency and course duration—could not be systematically extracted from the EHRs and analysed for their potential influence on the outcomes. Finally, a significant limitation inherent to the retrospective design is the lack of formal calibration among the numerous providers who performed the periodontal measurements during routine clinical care, which may have introduced inter‐examiner variability into the outcome data.

The clinical implications of these findings are significant. The results provide strong, real‐world evidence to support the clinician's decision to use adjunctive systemic antibiotics in the management of periodontitis, particularly in cases of greater severity. The clear indication is that patients with generalised severe periodontitis (e.g., Stage III/IV) are the subgroup most likely to derive a substantial clinical benefit. The consistent and superior performance of the amoxicillin + metronidazole combination reinforces its position as a primary regimen of choice when systemic antimicrobial therapy is indicated (Zandbergen et al. 2013). The demonstrated benefits on tooth mobility and furcation involvement are particularly compelling, as these factors are major determinants of long‐term tooth survival. Furthermore, our findings on the reduction of residual deep pockets are highly relevant; the greater PD reduction seen with combination therapy translates to a lower number of sites likely requiring subsequent surgical intervention. This evidence can help guide clinicians in developing treatment plans that not only arrest disease progression but also improve the stability of severely compromised teeth. From a clinical perspective, the reduction in the percentage of sites with PD ≥ 4 mm is perhaps the most significant finding.

Despite the clear benefits shown, these findings must be interpreted within the context of the global challenge of antibiotic resistance (Kapoor et al. 2012; Sun 2025). The results of this study should not be taken as a recommendation for the indiscriminate or routine use of antibiotics for all periodontitis patients. Instead, they provide evidence of efficacy that should be applied judiciously, targeting patients who are likely to receive the greatest benefit, such as those with deep pockets, extensive inflammation and signs of active disease that may not resolve with mechanical therapy alone (Kondreddy et al. 2025). Our stratified analysis helps define the patient population—those with severe, complex disease—who are most likely to receive a benefit that outweighs the potential risks. Prospective studies are also needed to evaluate the long‐term impact of these regimens on disease recurrence and the oral microbiome, which will be critical for developing more personalised and sustainable treatment protocols in the future.

## 5 Conclusion

Within the limitations of a retrospective study, the adjunctive use of amoxicillin + metronidazole provides a statistically significant and clinically robust benefit over SRP alone. The use of adjunctive amoxicillin + metronidazole provides the greatest clinical advantage in Stage III/IV periodontitis, while a statistically significant reduction in residual burden was noted in Stage I/II disease with a modest effect size. These data support a targeted antibiotic stewardship approach, where systemic agents are reserved specifically for severe cases to maximise clinical success and tooth stability while minimising unnecessary exposure.

## Author Contributions

Conception, acquisition of data, design: Georgios S. Chatzopoulos and Larry F. Wolff. Analysis, interpretation of results, drafting of the manuscript: Georgios S. Chatzopoulos. Critical review of the manuscript: Larry F. Wolff. Both authors approved the final version of the manuscript.

## Funding

The authors have nothing to report.

## Ethics Statement

The University of Minnesota Institutional Review Board (STUDY00016576) determined that this retrospective cohort study does not constitute research involving human subjects.

## Consent

Informed consent was waived due to the retrospective design of the study.

## Conflicts of Interest

The authors declare no conflicts of interest.

## Supporting information


**Data S1:** Supporting Information.

**Table S1:** Adjusted mean change in periodontal outcomes stratified by provider level (*N* = 3125).

## Data Availability

The data that support the findings of this study are available from the corresponding author upon reasonable request.
