# Supplementary material for: The Effect of Adjunctive Systemic Antibiotics on the Outcomes of Non‐Surgical Periodontal Therapy: A Retrospective Cohort Study
Source: J Clin Periodontol. 2026 Mar 16;53(5):719–34. doi: 10.1111/jcpe.70117 (PMC13086541; doi:10.1111/jcpe.70117)
Supplement: Supplementary file 1 — Data S1: Supporting Information. [file JCPE-53-719-s001.docx]

Supplementary Material and Methods

Study Design and Patient Cohort

This study was conducted as a retrospective cohort analysis using a large, de-identified electronic health records (EHR) database from multiple university dental clinics within the BigMouth Consortium. Data were collected electronically using standardized queries from the EHR database for the period between January 1, 2010, and December 31, 2020. The study cohort was comprised of patients with a diagnosis of periodontitis who received non-surgical periodontal therapy, specifically scaling and root planing (SRP), identified by the Current Dental Terminology (CDT) codes D4341 or D4342. The cohort selection process is detailed in Figure 1. All patient data was collected during routine clinical care and was fully anonymized prior to its inclusion in the research dataset. The study protocol was reviewed by the Institutional Review Board of the University of Minnesota and received a waiver of approval as it did not constitute research involving human subjects under federal definitions (STUDY00016576). Ethical clearance was additionally granted by the BigMouth Consortium for Oral Health Research and Informatics clinical review committee. All research activities were performed in accordance with the ethical principles of the Helsinki Declaration of 1975, as revised in 2013.

Data Extraction and Variable Definition

A comprehensive set of variables was extracted from the EHR for each patient meeting the inclusion criteria.

- Treatment Regimen: Patients were stratified into mutually exclusive groups based on their therapeutic regimen. The control group, "SRP Only," consisted of patients who received SRP without a concurrent systemic antibiotic prescription. The "SRP + Antibiotics" groups were defined as patients who received a prescription for a specific systemic antibiotic (Amoxicillin, Metronidazole, Doxycycline, Azithromycin, Clindamycin, Ciprofloxacin, or a combination of Amoxicillin and Metronidazole) on the day of or within 7 days following their SRP procedure date (a 0 to +7 day window).
  - Outcome Variables: Eight clinical outcomes were assessed by comparing measurements from the baseline ("Initial Exam") to the post-treatment ("Reevaluation") visit. The mean (SD) time to re-evaluation was 62 (14) days (range: 40-90 days).Patient-Level Outcomes: These included the change in: 1) Mean Probing Depth (MEAN_PD); 2) Mean Clinical Attachment Level (MEAN_CAL); 3) the percentage of sites with PD ≥ 4mm; and 4) the percentage of sites with Bleeding on Probing (BOP). Two additional composite endpoints were calculated to assess disease resolution: 5) "Residual Burden," defined as the percentage of sites with PD ≥ 4mm remaining at follow-up ; and 6) "Clinical Success," defined as the percentage of patients with ≤10% of sites having PD ≥ 4mm at follow-up.
  - Tooth-Level Categorical Outcomes: For individual teeth, the change in mobility and furcation was categorized as: 1) "Worsened" (an increase in the score/grade); or 2) "Not Worsened" (a decrease or no change in the score/grade).
- Covariates: To adjust for potential confounding, the following variables were extracted from the baseline examination: patient age, smoking status, sex, self-reported diabetes status, baseline disease severity, the number of teeth present at baseline, clinical provider level (DMD/DDS student, resident, or faculty), and the baseline value of the specific periodontal parameter being assessed as an outcome.

Statistical analysis

All statistical analyses were performed to compare the effectiveness of different adjunctive antibiotic regimens. First, descriptive statistics were used to characterize the baseline demographic and clinical features of the total study cohort, with continuous variables presented as means and standard deviations (SD) and categorical variables as frequencies and percentages.

For the five continuous, patient-level outcomes (PD reduction, CAL gain, reduction in % sites with PD ≥ 4mm, reduction in BOP, and Residual Burden), an Analysis of Covariance (ANCOVA) was used. Separate multivariable linear regression models were constructed for each outcome to calculate the adjusted mean change for each treatment regimen. For the binary patient-level outcome ("Clinical Success"), multivariable logistic regression models were utilized to calculate Adjusted Odds Ratios (aOR) and corresponding 95% confidence intervals.

All patient-level models controlled for the baseline value of the respective outcome variable and covariates including age, smoking status, sex, diabetes, number of teeth, and provider type. To evaluate comparative effectiveness, the models were first run with "SRP Only" as the reference group. Subsequently, direct head-to-head comparisons were performed between the combination therapy and all other antibiotic regimens, with p-values adjusted for multiple comparisons.

For the two tooth-level categorical outcomes (worsening of mobility and furcation), multilevel logistic regression models were used to calculate the odds of a tooth worsening, adjusting for the clustering of teeth within patients. A Pearson's Chi-squared test was also performed to compare the outcome distribution of each specific antibiotic regimen against the "SRP Only" reference group.

Additionally, a supplementary analysis was conducted to evaluate the influence of operator experience by comparing the adjusted mean clinical outcomes across the three provider categories: DMD/DDS student, resident, and faculty. These comparisons were adjusted for the same set of baseline covariates used in the primary analysis.

A stratified analysis was conducted based on baseline disease severity. Since formal staging was not available in the diagnostic codes (Tonetti et al., 2018), a proxy for the 2018 classification system was operationally defined for this study: patients with >30% of sites presenting with CAL ≥ 5mm were classified as having severe periodontitis (Stage III/IV proxy), while the remainder were classified as mild/moderate (Stage I/II proxy). The interaction between treatment group and disease severity was assessed for all primary outcomes.

A sensitivity analysis was also performed for deep pockets using a threshold of PD ≥ 5mm. Missing data for covariates was minimal (<2%). To address this, multiple imputation was performed using the Markov Chain Monte Carlo (MCMC) method with 5 imputations via SAS software. The imputation model included patient age, number of teeth, and the baseline values of the periodontal parameters. For all statistical tests, a p-value of less than 0.05 was considered statistically significant.
