# Supplementary material for: The Effect of Adjunctive Systemic Antibiotics on the Outcomes of Non‐Surgical Periodontal Therapy: A Retrospective Cohort Study
Source: J Clin Periodontol. 2026 Mar 16;53(5):719–34. doi: 10.1111/jcpe.70117 (PMC13086541; doi:10.1111/jcpe.70117)
Supplement: Supplementary file 2 — Table S1: Adjusted mean change in periodontal outcomes stratified by provider level (N = 3125). [file JCPE-53-719-s002.docx]

Supplementary Table 1: Adjusted Mean Change in Periodontal Outcomes Stratified by Provider Level (N = 3,125)

| Provider Category | N | Change in Mean PD (mm) [95% CI] | Change in Mean CAL (mm) [95% CI] | Change in % Sites with PD ≥ 4mm [95% CI] | Change in % Sites with BOP [95% CI] |
| --- | --- | --- | --- | --- | --- |
| DMD/DDS Student | 2,156 | -0.55 [-0.60, -0.50] | -0.42 [-0.48, -0.36] | -14.5 [-15.2, -13.8] | -21.5 [-22.5, -20.5] |
| Resident | 781 | -0.62 [-0.68, -0.56] | -0.50 [-0.58, -0.42] | -16.2 [-17.5, -14.9] | -24.1 [-25.8, -22.4] |
| Faculty | 188 | -0.60 [-0.72, -0.48] | -0.48 [-0.62, -0.34] | -15.8 [-18.1, -13.5] | -23.5 [-26.1, -20.9] |
| p-value |  | 0.125 | 0.085 | 0.042 | 0.031 |

Abbreviations: N, Number of patients; CI, Confidence Interval; PD, Probing Depth; CAL, Clinical Attachment Level; BOP, Bleeding on Probing; DMD, Doctor of Dental Medicine.

 p-values represent the significance of the difference between provider categories, derived from the ANCOVA model adjusting for baseline characteristics.
